# Coefficient of variation as an image-intensity metric for cytoskeleton bundling

**DOI:** 10.1038/s41598-020-79136-x

**Published:** 2020-12-21

**Authors:** Takumi Higaki, Kae Akita, Kaoru Katoh

**Affiliations:** 1 International Research Organization for Advanced Science and Technology, Kumamoto University, 2-39-1 Kurokami, Chuo-ku, Kumamoto, Japan; 2 Department of Chemical Biological Science, Faculty of Science, Japan Women’s University, 2-8-1 Mejirodai, Bunkyo-ku, Tokyo, Japan; 3 Biomedical Research Institute, National Institute of Advanced Industrial Science and Technology (AIST), Tsukuba, Japan

**Keywords:** Cell biology, Computational biology and bioinformatics

## Abstract

The evaluation of cytoskeletal bundling is a fundamental experimental method in the field of cell biology. Although the skewness of the pixel intensity distribution derived from fluorescently-labeled cytoskeletons has been widely used as a metric to evaluate the degree of bundling in digital microscopy images, its versatility has not been fully validated. Here, we applied the coefficient of variation (CV) of intensity values as an alternative metric, and compared its performance with skewness. In synthetic images representing extremely bundled conditions, the CV successfully detected degrees of bundling that could not be distinguished by skewness. On actual microscopy images, CV was better than skewness, especially on variable-angle epifluorescence microscopic images or stimulated emission depletion and confocal microscopy images of very small areas of around 1 μm^2^. When blur or noise was added to synthetic images, CV was found to be robust to blur but deleteriously affected by noise, whereas skewness was robust to noise but deleteriously affected by blur. For confocal images, CV and skewness showed similar sensitivity to noise, possibly because optical blurring is often present in microscopy images. Therefore, in practical use with actual microscopy images, CV may be more appropriate than skewness, unless the image is extremely noisy.

## Introduction

Bundling of the cytoskeleton, which consists of actin filaments and microtubules, is a critical step in the formation of the high-order structures that are tightly related to cellular activities including cell division^1–4^, cell morphogenesis^5,6^, and cell motility^7–9^. Microscopic assessment of cytoskeletal bundling is a fundamental experimental method in the field of cell biology. In terms of experimental throughput and data objectivity, quantitative methods based on image analysis of digital microscopy are clearly superior to qualitative judgments based on a researcher’s visible inspection. In cells with fluorescently labeled cytoskeletons, cytoskeleton bundles show stronger fluorescence than single filaments, suggesting that bundling level could be quantitatively evaluated by measuring the absolute values of fluorescent intensity peaks in fluorescent microscopy images^10^. However, this method may not be accurate in some cases, such as when the abundance levels of cytoskeleton fluorescent protein markers differ according to cell status^11^. We previously reported a robust quantification method for measuring changes in the abundance levels of fluorescent protein markers^7,12^, which uses the skewness of the pixel intensity distribution derived from the cytoskeleton as a metric for the quantitative evaluation of actin filament bundling in plant stomatal guard cells. Our ‘skewness method’ has been used widely as a standard method for the quantitative detection of bundling of actin filaments and microtubules^13^, not only in plant cells^14–17^, but also in vitro^18–20^, and in fission yeast^21^, fungi^22^, and mammalian cells^23,24^.

Nonetheless, the versatility of the skewness method has not been fully validated; for example, its tolerance to image blur and noise has not been determined. In addition, the skewness method may be unsuitable and misleading in some cases, such as when cytoskeletons are extremely bundled in an image. We first noticed that cytoskeletal bundling could be quantitatively evaluated according to the skewness of the intensity distribution (a statistic indicating the lack of Gaussian normality) when analyzing the intensity histograms of skeletonized images of *Arabidopsis thaliana* guard cells expressing the actin filament marker GFP-actin binding domain 2 (GFP-ABD2) fusion protein (Supplemental Fig. 1)^7^. We observed the Gaussian intensity distributions of the major peaks of single actin filaments, and found that higher-intensity bundles had skewed intensity distributions, resulting in high skewness values. Indeed, these skewness values were successfully used to detect pharmacologically-induced actin bundling in guard cells treated with the actin filament bundling agent 2,3,5-triiodobenzoic acid (TIBA; Supplemental Fig. 1)^7^. However, if most filaments are in bundles, there will be very few single filaments. In such a case, the major intensity distribution will shift towards higher values indicating increased brightness, which would reduce skewness. To ensure rigorous evaluation of cytoskeleton bundling, the versatility of the skewness method should be considered before using it, and alternate metrics are needed in cases where the skewness method is inappropriate.

The coefficient of variation (CV) is a measure of relative variability, which is the ratio of the standard deviation to the mean. Here, we examined CV as an alternative image-intensity metric for the evaluation of cytoskeleton bundling, investigating whether bundling could also be detected by widening the intensity distribution of the cytoskeletal pixels (Supplemental Fig. 1). To validate the versatility of skewness and CV methods as bundling indicators, we used various types of cytoskeleton images to compare them. In many cases, the CV method allowed successful detection of cytoskeleton bundling with equal or greater sensitivity than the skewness method, and it was particularly useful for blurred images where skewness was inappropriate.

## Results

### Evaluation of bundling using synthetic images

**Figure 1:**
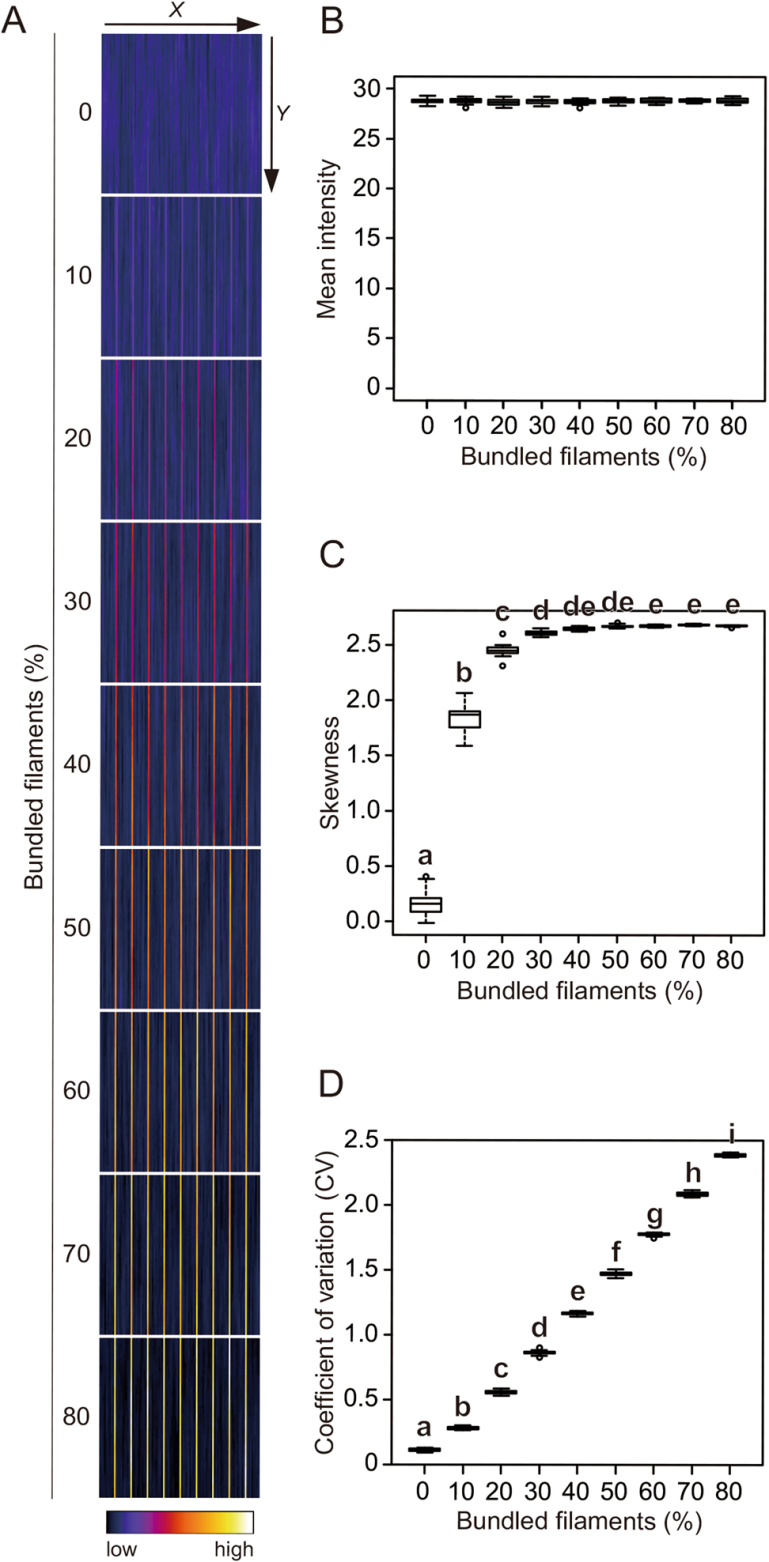
Evaluation of filament bundling in synthetic images. (**A**) Representative synthetic images of virtual cytoskeleton bundling. The images were built by adding filaments with constant intensity and length at random positions. To mimic bundles, the positions of the filaments on the *X*-coordinate were restricted to 1/20 for different percentages of the filaments (0–80%). (**B**) Mean intensity of the images. (**C**) Skewness of the intensity distribution. (**D**) CV of the intensity distribution. Significance was tested using the Tukey–Kramer test (*P* < 0.01). Different lowercase letters indicate significant differences. N = 20.

To analyze skewness and CV properties, we first used synthetic images that mimic cytoskeleton bundling as ideal images without optical blur or camera noise. The synthetic images were created by the virtual placement of the same number of filaments with the same orientation, length, and intensity at random positions in a zero-background image (Fig. 1A, 0%). To simulate bundling of the filaments, the position of a certain percentage of filaments (corresponding to ‘Bundled filaments (%)’ in Fig. 1A) was constrained in the axis perpendicular to the fiber (i.e., the *X*-coordinate in Fig. 1A, 10%–80%, Supplemental Fig. 2). We confirmed that the average intensity of the images was almost the same, regardless of the spatial arrangement of the filaments (i.e. degrees of bundling) (Fig. 1B). The skewness and CV values of the synthetic images showed different patterns according to the different proportions of bundled filaments (Fig. 1C, D, Supplemental Fig. 3). For skewness, the sensitivity values increased with bundling up to a proportion of 30% of bundled filaments, then leveled off for higher proportions of bundled filaments (Tukey–Kramer test, *P* < 0.01; Fig. 1C). For CV, the sensitivity values increased with increasing proportions of bundled filaments and all degrees of bundling were distinguished (Tukey–Kramer test, *P* < 0.01; Fig. 1D). These results suggest that CV, rather than skewness, may be a more appropriate numerical indicator for evaluating bundling when the proportion of bundled filaments is high.

### Evaluation of bundling using actual microscopy images

We examined the properties of skewness and CV using synthetic images in which the average intensity was almost the same between images, regardless of the bundling level (Fig. 1). However, in actual cells, the cytoskeleton bundling state may affect the cytoskeleton polymerization rate. In addition, synthetic images differ from actual microscopy images in that they are not subject to disturbances such as optical blur and camera noise. Therefore, we next analyzed the skewness and CV properties of actual cytoskeleton images. The microscopy images of cytoskeleton bundles were acquired using different materials and microscopes. To eliminate the effects of background signals, all images were skeletonized before measuring the skewness and CV of the intensity distributions of the pixels representing the cytoskeletons^12^, as shown in Supplemental Fig. 1 (see also Materials and Methods).

**Figure 2:**
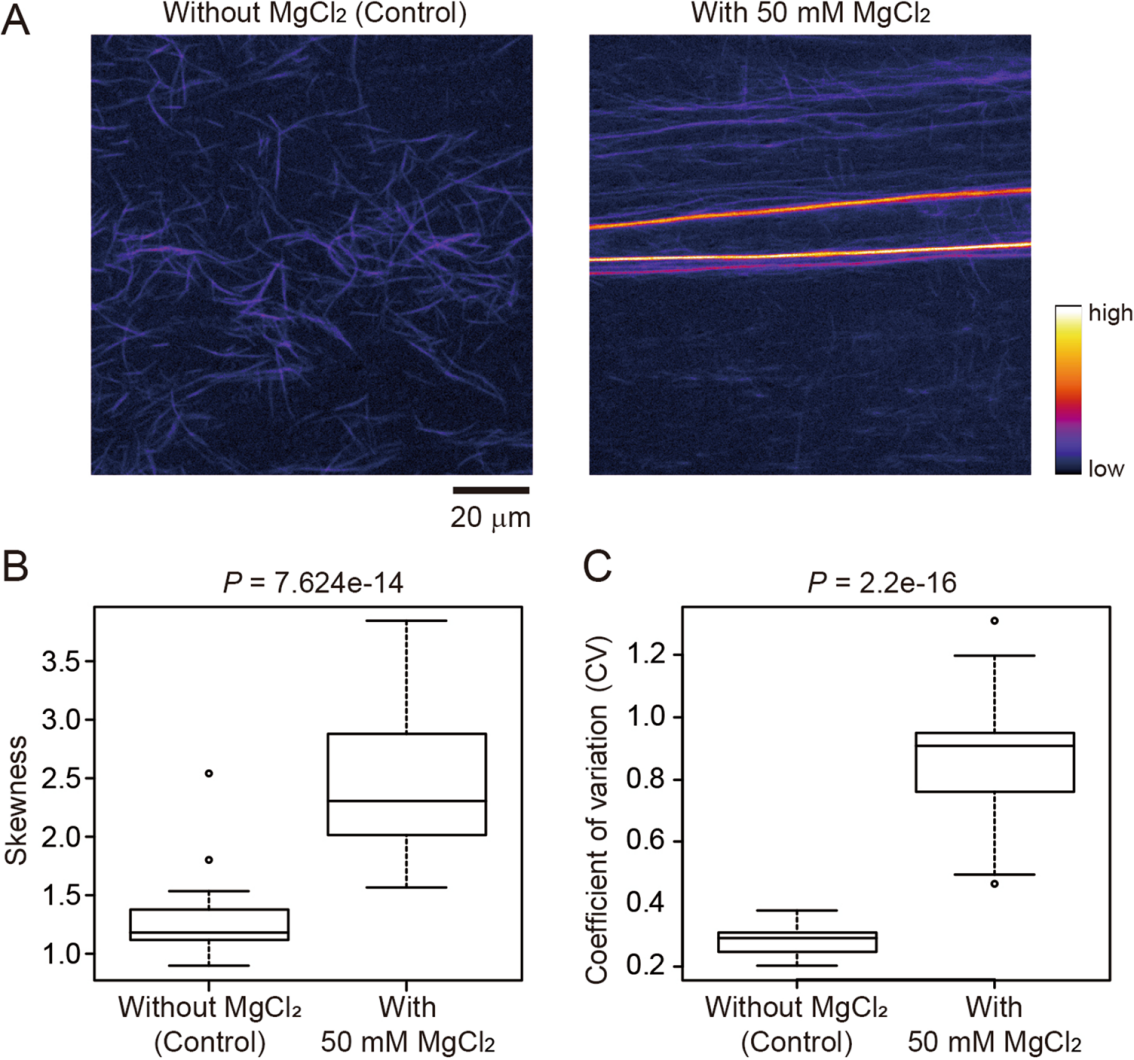
Evaluation of actin filament bundling in vitro. (**A**) Representative confocal laser scanning microscopy images of in vitro ATTO 390-labeled actin filaments. Control images (left) and those with bundling due to the addition of MgCl_2_ (right) are shown. Scale bar indicates 20 μm. (**B**) Skewness of the intensity distribution. (**C**) CV of intensity distribution. Significance was determined using the Mann–Whitney U-test. N = 30.

First, we used confocal laser scanning microscopy (CLSM) to capture actin filaments and bundles in an in vitro actin polymerization system. The actin filaments, which were labeled with the fluorescent dye ATTO 390, were induced into a highly bundled state by the addition of 50 mM MgCl_2_ (Fig. 2A, Supplemental Fig. 4A). The skewness and CV values of the images with and without MgCl_2_ were significantly different (Mann–Whitney U-test, *P* = 7.624e−14 for skewness and *P* = 2.2e−16 for CV; Fig. 2B, C, Supplemental Fig. 4B). Therefore, the skewness and CV methods both successfully detected the MgCl_2_-induced bundling of actin filaments in vitro.

**Figure 3:**
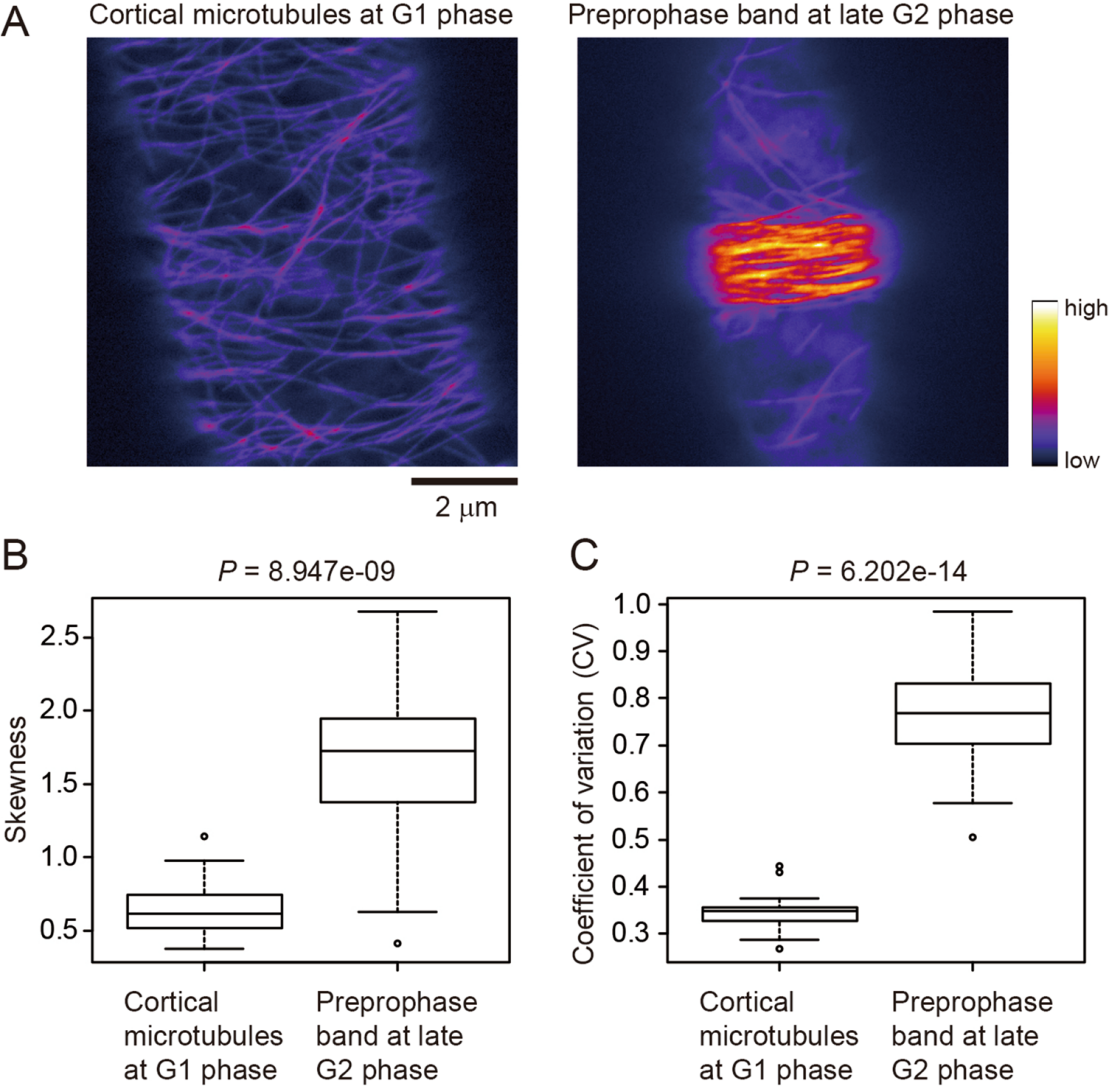
Evaluation of microtubule bundling in vivo. (**A**) Representative confocal laser scanning microscopy images of yellow fluorescent protein (YFP)-tubulin-labeled microtubules in tobacco BY-2 cells. Cortical microtubules at the G1 phase (left) and highly bundled cortical microtubules in the preprophase band (right) are shown. Scale bar indicates 2 μm. (**B**) Skewness of the intensity distribution. (**C**) CV of the intensity distribution. Significance was tested using the Mann–Whitney U-test. N = 24.

Second, we analyzed preprophase bands (PPBs) as an example of high-bundling of microtubules under in vivo physiological conditions. PPBs contain bundles of cortical microtubules that localize just beneath the plasma membrane of plant cells during the late G2 phase and are thought to contribute to determination of the cell division plane^25^. CLSM images of cortical microtubules at the G1 phase and PPBs at the late G2 phase were obtained with cell cycle synchronization of tobacco BY-2 cells stably expressing yellow fluorescent protein (YFP)-tagged tubulin^26^ (Fig. 3A, Supplemental Fig. 5A). Both skewness and CV successfully detected microtubule bundling during PPB formation, with the skewness and CV values for differences between the images with and without bundling being highly significant, but more so for CV (Mann–Whitney U-test, *P* = 8.947e−09 for skewness and *P* = 6.202e−14 for CV; Fig. 3B, C, Supplemental Fig. 5B).

**Figure 4:**
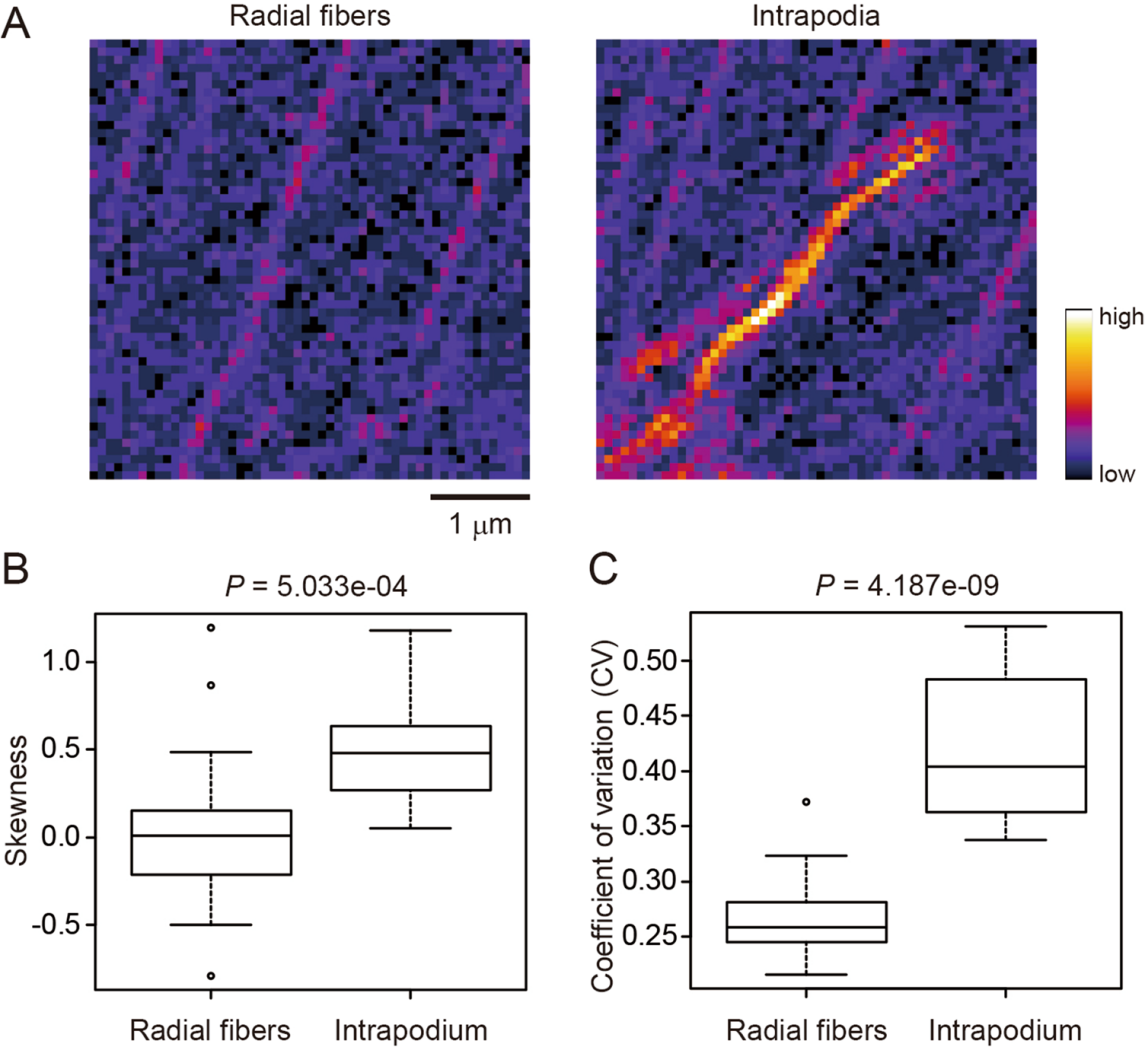
Evaluation of actin bundling in Pol-Scope images. (**A**) Representative Pol-Scope images of radial actin fibers (left) and intrapodium composed of highly bundled actin filaments (right) in the living and unstained growth cone of *Aplysia* bag cell neurons. Scale bar indicates 1 μm. (**B**) Skewness of the intensity distribution. (**C**) CV of the intensity distribution. Significance was tested using the Mann–Whitney U-test. N = 18.

Third, to visualize birefringence retardation, we tested images obtained by modified polarized light microscopy using a Pol-Scope^27,28^. The Pol-Scope was shown to be useful for visualizing aligned actin filaments including radial actin fibers and intrapodium composed of actin bundles in the living and unstained growth cone of *Aplysia* bag cell neurons^27^ (Fig. 4A, Supplemental Fig. 6A). As in the case of PPB images (Fig. 3), both skewness and CV values successfully detected actin bundling in intrapodium, but more so for CV (Mann–Whitney U-test, *P* = 5.033e−04 for skewness and *P* = 4.187e−09 for CV; Fig. 4B, C, Supplemental Fig. 6B).

**Figure 5:**
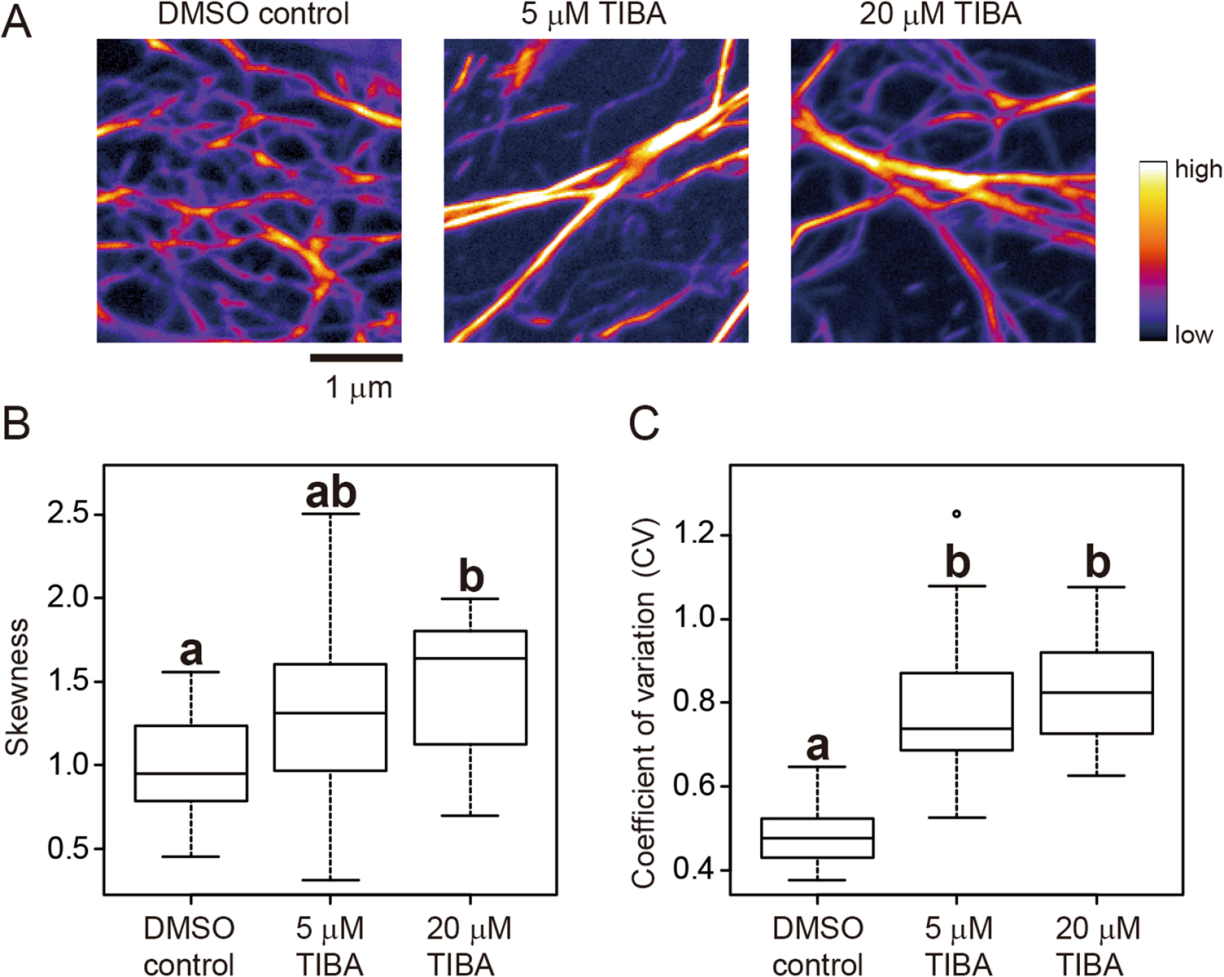
Evaluation of actin filament bundling in confocal laser scanning microscopy (CLSM) images. (**A**) Representative CLSM images of GFP-ABD2-labeled actin filaments in hypocotyl cells of *A. thaliana* plants. Actin filaments treated with DMSO (control), and 5 μM and 20 μM TIBA, an actin filament bundling agent, are shown. Scale bar indicates 1 μm. (**B**) Skewness of the intensity distribution. (**C**) CV of the intensity distribution. Significance was tested using the Tukey–Kramer test (*P* < 0.01). Different lowercase letters indicate significant differences. N = 24.

**Figure 6:**
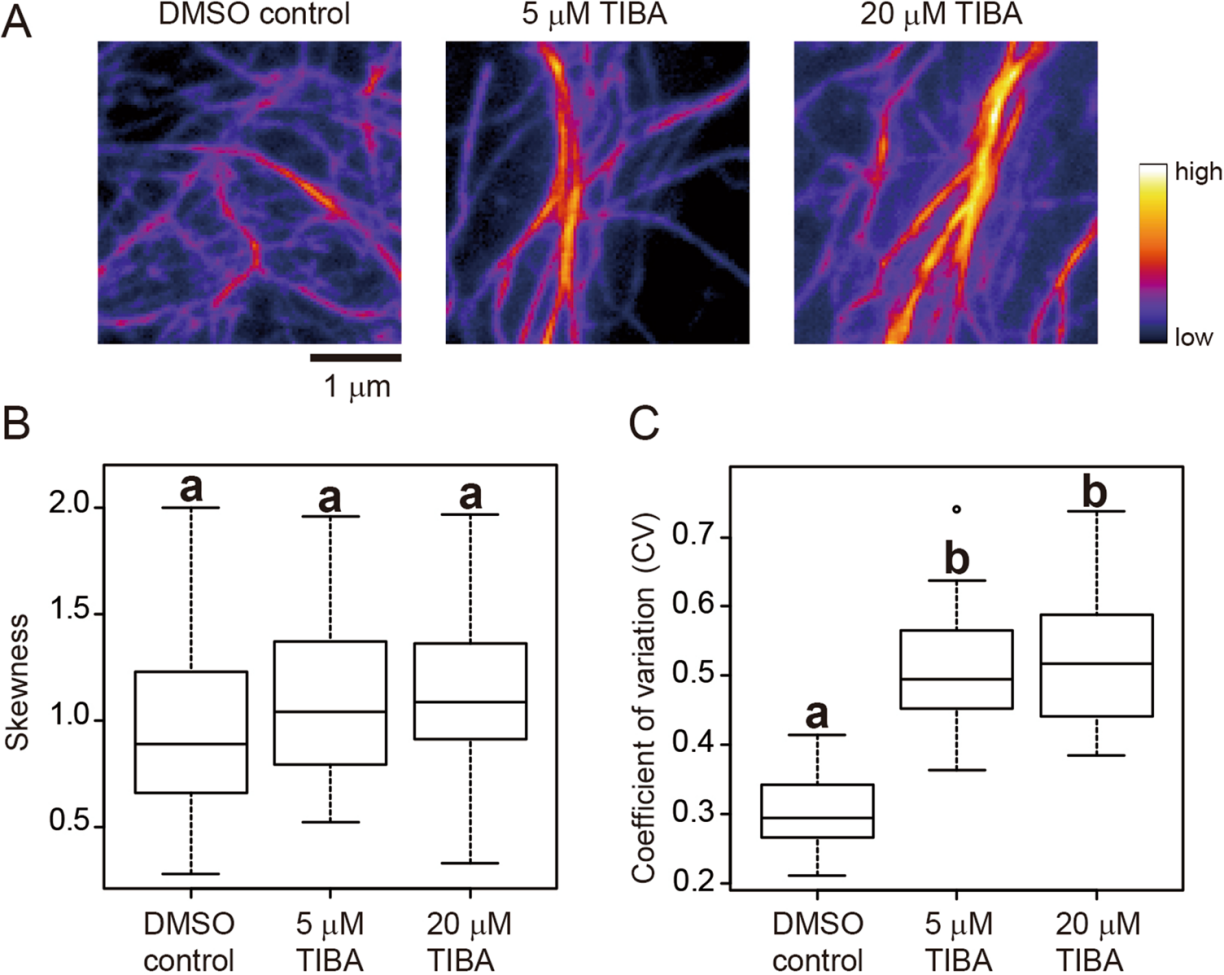
Evaluation of actin filament bundling in variable-angle epifluorescence microscopy (VAEM) images. (**A**) Representative VAEM images of GFP-ABD2-labeled actin filaments in hypocotyl cells of *A. thaliana* plants. Actin filaments treated with DMSO (control), and 5 μM and 20 μM TIBA, an actin filament bundling agent, are shown. Scale bar indicates 1 μm. (**B**) Skewness of the intensity distribution. (**C**) CV of intensity distribution. Significance was tested using the Tukey–Kramer test (*P* < 0.01). Different lowercase letters indicate significant differences. N = 24.

Fourth, we examined cytoskeleton images taken by variable-angle epifluorescence microscopy (VAEM) using a total internal reflection microscope^29^. We used VAEM because it is better than CLSM for monitoring rapid movement of fluorescently-labeled cytoskeletons at cell surfaces^15,30–32^. For comparison, we used *A. thaliana* hypocotyl epidermal cells expressing GFP-ABD2 and obtained cortical actin filament images by both CLSM and VAEM. We measured the skewness and CV of the CLSM or VAEM images of cells treated without (DMSO control) or with 5 or 20 μM TIBA^7,33^ (Figs. 5A, 6A, Supplemental Figs. 7A, 8A). Statistically significant differences between the image-intensity values were determined using the Tukey–Kramer test (*P* < 0.01). For the CLSM images, the differences in skewness values were statistically significant between the DMSO control and the 20 μM TIBA treatment, but not between the control and 5 μM TIBA treatment, or between the 5 μM and 20 μM TIBA treatments (Fig. 5B, Supplemental Fig. 7B). For the CLSM images, the differences in CV values were statistically significant between the DMSO control and the 5 μM and 20 μM TIBA treatments, but not between the 5 μM and 20 μM treatments (Fig. 5C, Supplemental Fig. 7B). For the VAEM images, no significant differences in skewness values were detected between the three samples (Fig. 6B, Supplemental Fig. 8B), whereas the differences in CV values were statistically significant between the DMSO control and 5 μM and 20 μM TIBA treatments (Fig. 6C, Supplemental Fig. 8B), as was also the case for the CLSM images (Fig. 5C, Supplemental Fig. 7B). These results suggest that CV is more suitable than skewness for evaluating cytoskeleton bundling in VAEM images.

**Figure 7:**
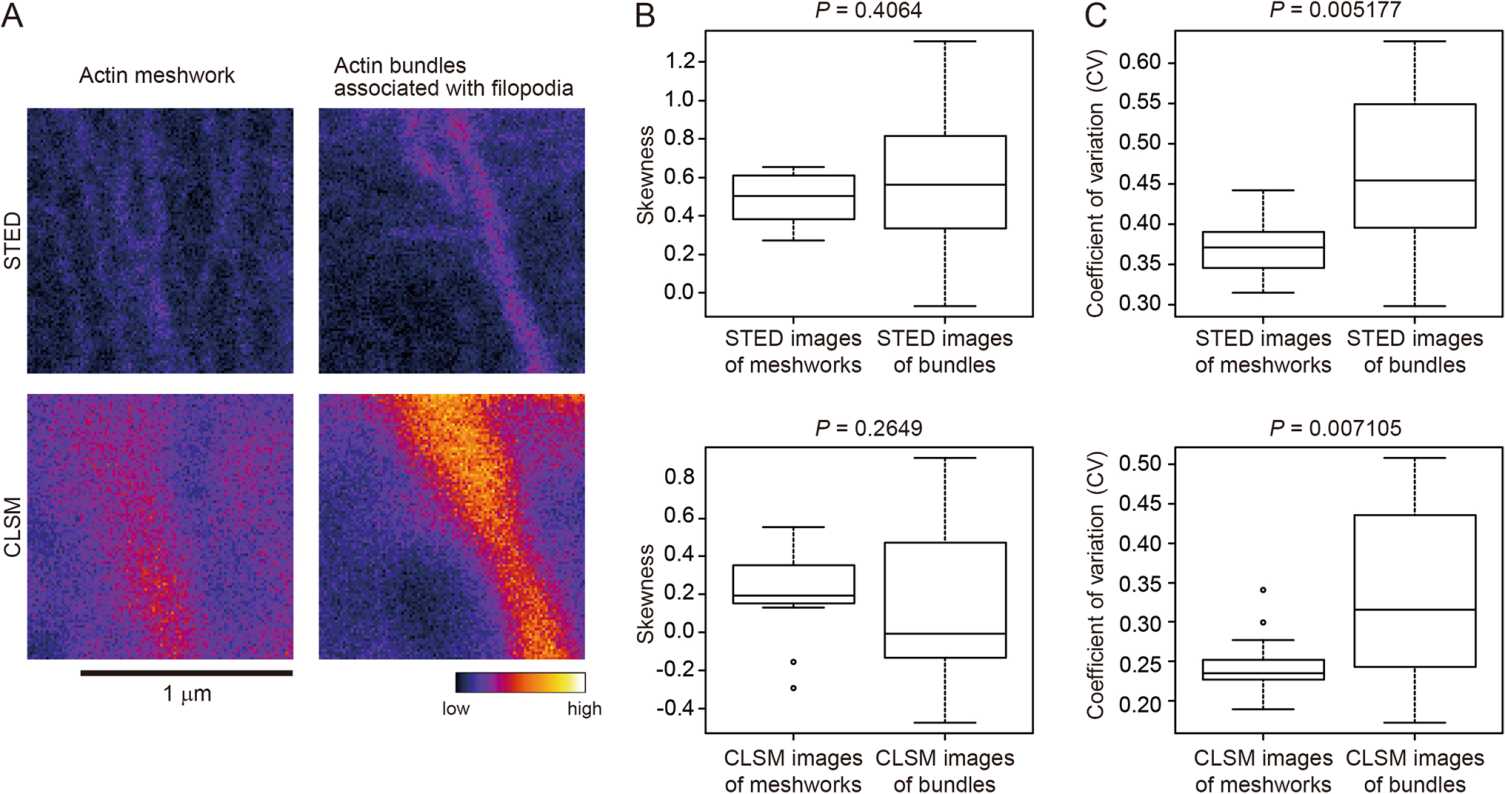
Evaluation of actin filament bundling in STED and CLSM images. (**A**) Representative stimulated emission depletion microscopy (STED) and confocal laser scanning microscopy (CLSM) images of the same regions of TRITC-phalloidin-labeled actin filaments in NG108-15 cells. The actin meshwork and actin filament bundles associated with filopodia are shown. Scale bar indicates 1 μm. (**B**) Skewness of the intensity distribution. (**C**) CV of the intensity distribution. Significance was determined using the Mann–Whitney U-test. N = 18.

Fifth, we examined images obtained by super-resolution microscopy. The cytoskeleton is often used as an example to demonstrate the power of super-resolution microscopy, and the use of super-resolution microscopy is expected to increase in future cytoskeleton studies. Among the available super-resolution microscopy technologies, we focused on stimulated emission depletion microscopy (STED)^34^ because it can be operated seamlessly with CLSM. A conventional 2D STED system was used to achieve the same *Z*-axis resolution for STED and CLSM. We stained actin filaments in NG108-15 cells (a mouse neuroblastoma-rat glioma hybrid cell line) with TRITC-phalloidin, then acquired STED and CLSM images of the actin meshwork and bundles associated with filopodia in the same very small area of around 1 μm^2^ (Fig. 7A, Supplemental Fig. 9A,B). For the STED and CLSM images, no significant differences in skewness values were detected between the actin meshwork and bundles (Mann–Whitney U-test, *P* = 0.4064 for STED and *P* = 0.2649 for CLSM; Fig. 7B, Supplemental Fig. 9C,D). Conversely, the differences in CV values between the actin meshwork and the bundles in the very small regions were statistically significant with both STED and CLSM (Mann–Whitney U-test, *P* = 0.005177 for STED and *P* = 0.007105 for CLSM; Fig. 7C, Supplemental Fig. 9C,D).

### Effects of image blur and noise

**Figure 8:**
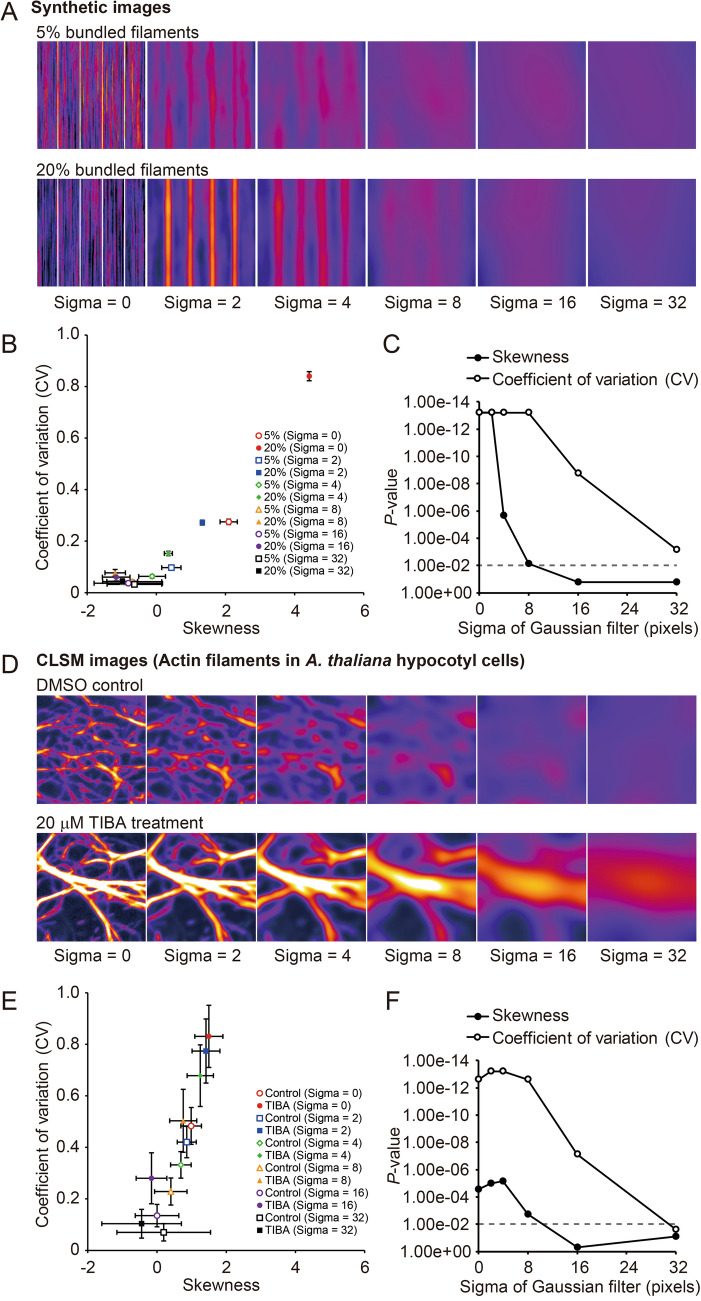
Effects of blur on evaluations of cytoskeleton bundling in synthetic images and actual CLSM images. (**A**) Original and blurred synthetic images of virtual cytoskeleton bundling. Gaussian filters with different sigma values (0–32 pixels) were applied to the images with 5% and 20% bundled filaments. (**B**) A scatter plot between skewness and CV. (**C**) Scatter plot showing the sensitivity of the skewness and CV methods for detecting bundles in the blurred synthetic images. *P* values between the images with 5% and 20% bundled filaments were determined using the Mann–Whitney U-test The broken line indicates the significance level (*P* = 0.01). (**D**) Original and blurred confocal laser scanning microscopy (CLSM) images of GFP-ABD2-labeled actin filaments in hypocotyl cells of *A. thaliana* plants. Gaussian filters with different sigma values (0–32 pixels) were applied to the DMSO control and 20 μM TIBA treatment images. (**E**) Scatter plot between skewness and CV. (**F**) Scatter plot showing the sensitivity of the skewness and CV methods in detecting bundles in the blurred CLSM images. *P* values were determined using the Mann–Whitney U-test. The broken line indicates the significance level (*P* = 0.01).

For the actual microscopy images, skewness was not as good as CV at detecting cytoskeletal bundling in VAEM images (Fig. 6), or in STED or CLSM images of very small areas (about 1 μm^2^; Fig. 7). To explore the reasons for the different detection sensitivities of skewness and CV, we degraded the synthetic and actual images by adding blur and noise, and then analyzed the robustness of skewness and CV to this image degradation. To blur the images, we applied a Gaussian filter with different sigma values (0, 2, 4, 8, 16, and 32 pixels) to some of the synthetic images in Fig. 1 (5% and 20% of bundled filaments; Fig. 8A) and some of the CLSM images of actin filaments in *A. thaliana* hypocotyl cells in Fig. 5 (DMSO control and 20 μM TIBA treatment; Fig. 8D). For the synthetic images, both skewness and CV detected bundles with high sensitivity in images blurred with a Gaussian filter with a sigma value of up to 2; however, at higher sigma values, the sensitivity of the skewness method decreased more rapidly than that of the CV method (Fig. 8B, C). For the actual CLSM images without Gaussian filtering, CV was better at detecting bundles than skewness (Fig. 8E, F), and because of this higher baseline sensitivity of CV, it performed better than skewness at all the sigma values, although the sensitivity of both methods dropped for sigma values above 4 (Fig. 8F, F).

**Figure 9:**
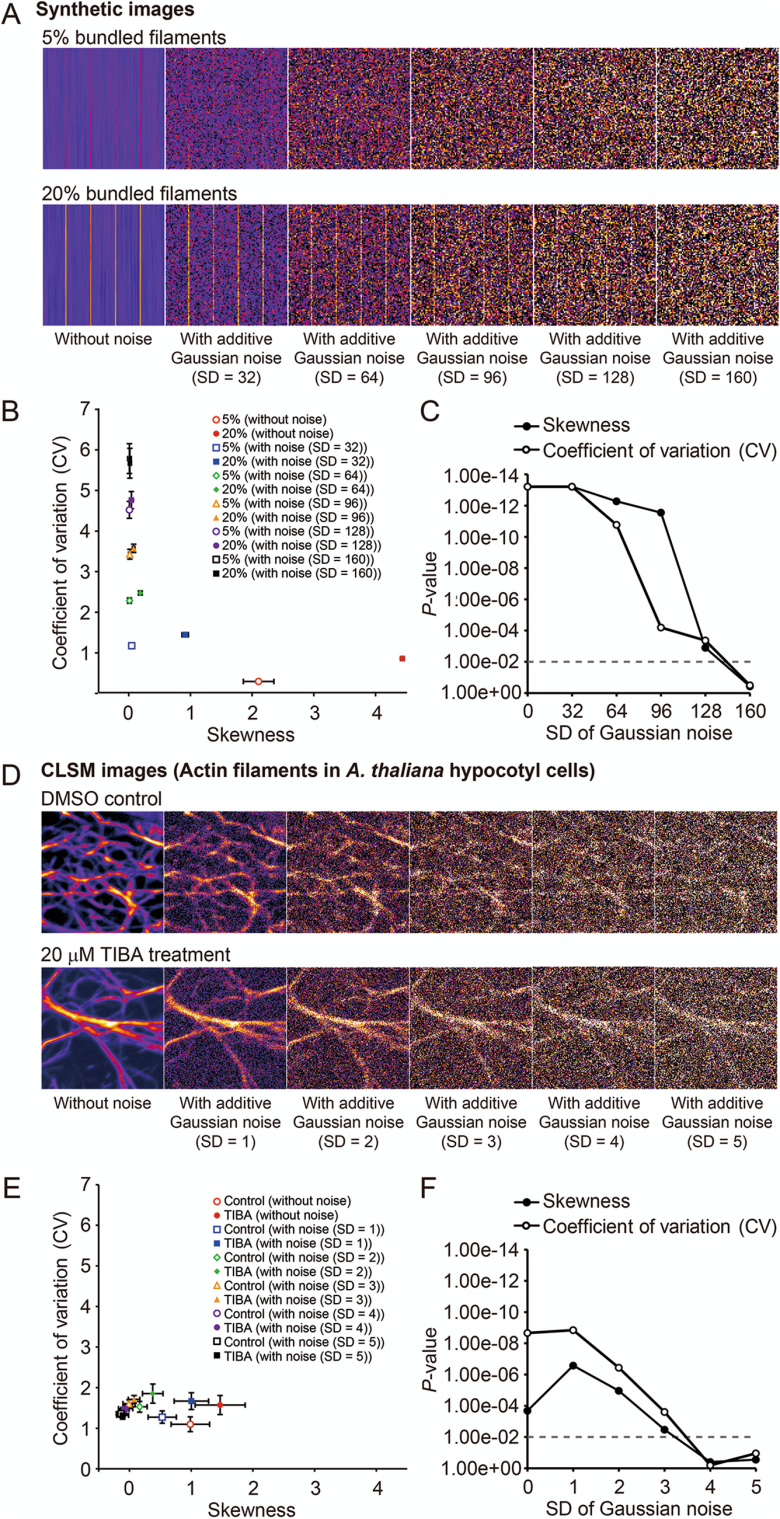
Effects of noise on evaluations of cytoskeleton bundling in synthetic images and actual CLSM images. (**A**) Original and synthetic images of virtual cytoskeleton bundling with random Gaussian noise. Random Gaussian noise [average intensity = 28, standard deviation (SD) = 32–160] was added to the images with 5% and 20% bundled filaments. (**B**) Scatter plot between skewness and CV. (**C**) Scatter plot showing the sensitivity of the skewness and CV methods in the detection of bundles in synthetic images with added noise. *P* values between the images with 5% and 20% bundled filaments were determined using the Mann–Whitney U-test. The broken line indicates the significance level (*P* = 0.01). (**D**) Original and confocal laser scanning microscopy (CLSM) images of GFP-ABD2-labeled actin filaments in hypocotyl cells of *A. thaliana* plants. The image intensity was first normalized (average intensity = 0, standard deviation (SD) = 1), then Gaussian noise (average intensity = 0, SD = 1–5) was added to the actin filaments treated with DMSO (control images) and 20 μM TIBA (treatment images). Before measuring the skewness and CV, the images were skeletonized. (**E**) Scatter plot between skewness and CV. (**F**) Scatter plot showing the sensitivity of the skewness and CV methods in the detection of bundles in the CLSM images with added noise. *P* values were determined using the Mann–Whitney U-test. The broken line indicates the significance level (*P* = 0.01).

Noise was added to the synthetic images (5% and 20% of the bundled filaments; Fig. 9A) using random Gaussian noise with an average intensity of 28.0 and standard deviations (SDs) of 32, 64, 96, 128, and 160. For the CLSM images of actin filaments in *A. thaliana* hypocotyl cells (DMSO control and 20 μM TIBA treatment), the image intensity was normalized (average intensity = 0, SD = 1), then random Gaussian noise with an average intensity of 0 and SDs of 1, 2, 3, 4, and 5 (Fig. 9D) was applied. For the synthetic images, skewness was more robust to the addition of noise with SD of 64 and 96 than was CV (Fig. 9B, C). For the CLSM images, the sensitivity of both skewness and CV decreased equally as the SD of the noise increased, although the baseline sensitivity was higher for CV (Fig. 9E, F). These results suggest that in the evaluation of cytoskeleton bundling, skewness was robust to noise and CV was robust to image blur.

## Discussion

Quantitative evaluation is an essential way to examine cytoskeleton organization. Various tools and algorithms are available to quantify cytoskeleton spatial features, including orientation^35^, distribution^36^, network pattern^37,38^, and time-evolution^39,40^. These methodologies have greatly contributed to the understanding of cytoskeleton organization and dynamics. Furthermore, we have shown that intensity features are also useful for understanding cytoskeleton organization, especially cytoskeleton bundling. We previously reported that skewness of the intensity distribution could be used to detect actin filament bundling in plant stomatal guard cells^7^, and the skewness method is now commonly used to detect bundling of both actin filaments and microtubules^13^. However, we suspected that skewness may not be suitable for evaluating extreme bundling conditions when there are not enough single filaments. To investigate this further, we applied another image-intensity statistic, CV, as a numerical indicator of cytoskeleton bundling, and compared the performance of skewness and CV using various types of cytoskeleton images. Because CV indicates variation (i.e., the ratio of the standard deviation to the mean) in the intensity values of cytoskeleton pixels and is independent of the shape of the distribution, we considered that it would allow detection of bundles even in the absence of sufficient single filaments. As expected, the evaluation using synthetic images showed that CV successfully detected the degree of bundling in extremely bundled conditions that could not be distinguished by skewness (Fig. 1). CV is a standardized metric with average values, and is therefore supposed to be robust to changes in the abundance of fluorescent protein markers^11^, similar to skewness. However, theoretically, if the intensities of the bundles are homogeneous (i.e., if the number of filaments to be bundled is fixed), the intensity variability would be so small that CV could not evaluate the bundling. Nevertheless, CV is expected to be a valuable and practical metric to detect cytoskeletal bundling because such homogeneous bundling is rarely observed in actual cytoskeleton images, at least in the in vitro and in vivo systems used in this study (Figs. 2–7).

We also examined the effects of image blur and noise on the abilities of skewness and CV to detect cytoskeletal bundles. For the synthetic images, CV was shown to be robust to blur but more affected by noise, whereas skewness was affected by blurring but was robust to noise (Figs. 8A–C, 9A–C). For the actual CLSM images, CV was more robust than skewness to blur (Fig. 8D–F), while the sensitivity of CV to noise was more than that of skewness (Fig. 9D–F), possibly because the actual microscope images were subject to optical blur. These results suggest that CV is better than skewness in practical operations with actual microscopy images, unless the image is subject to extreme noise. Indeed, skewness failed to detect actin bundles in VAEM images, which are more prone to blur than CLSM images^29^, whereas CV succeeded (Fig. 6). CV was also better than skewness in detecting cytoskeleton bundling in the STED and CLSM images of very small areas of around 1 μm^2^ (Fig. 7). Interestingly, no differences in detection sensitivity were found between STED and CLSM images for either skewness or CV, even though the STED images were clearly of higher resolution than the CLSM images (Fig. 7B, C). This finding may be because of the inevitable decrease in signal intensity due to the improved spatial resolution in STED^41^ (Fig. 7A). Thus, because the detection sensitivities of both skewness and CV are reduced by noise (Fig. 9D–F), even if the spatial resolution is increased by STED (i.e., blur is reduced), the detection sensitivities are not necessarily improved on STED in comparison with CLSM. These results suggest that CV can detect cytoskeleton bundling in blurred images when skewness fails. Therefore, CV may reveal overlooked findings through data mining of previously published high-throughput screening of image datasets obtained by conventional fluorescence microscopy^42–44^.

In conclusion, to quantitatively evaluate cytoskeleton bundling, we applied two image-intensity statistics to cytoskeleton pixels, the previously applied metric of skewness and the new metric of CV. Skewness of the intensity distribution has been shown to be useful for the quantitative evaluation of cytoskeletal bundling, possibly because most cytoskeleton images have a sufficient amount of single filaments; however, caution is necessary when the filaments are excessively bundled or when the images are blurred. We showed that for all the image sets used in this study, the CV of the intensity values of the cytoskeleton can quantitatively evaluate bundling with a sensitivity equal to or greater than that of skewness. CV was not suitable for analysis of noisy images, but was suitable for the analysis of excessive bundling or blurred images, images for which skewness failed. Thus, CV can complement skewness. Furthermore, these image-intensity statistics can also be used to quantify various biological or chemical phenomena other than cytoskeletal bundling, with the statistics having been used to examine spectral data in the photobiology field^45^. Indeed, it was reported that the aggregation of nanoparticles in a suspension was detected by skewness applied to variance spectroscopy^46^. Image-intensity statistics, including skewness and CV, are simple metrics with low computational load and are useful in multiple types of biochemical image evaluations. We believe that both skewness and CV will be applied to quantitative high-throughput analysis in many research directions.

## Methods

### Materials and microscopy

In vitro actin polymerization and MgCl_2_-induced bundling of actin filaments were performed using Actin-Toolkit Fluorescence Microscopy (ATTO390-Actin; Hypermol EK, Bielefeld, Germany) according to the manufacturer’s protocols. The images of in vitro actin filaments were obtained with a CLSM (FV3000; Olympus, Tokyo, Japan) with a 405-nm laser.

Cytoskeletons in plant cells were captured using a transgenic line of cultured tobacco BY-2 cells stably expressing YFP-tubulin^26^, and *A. thaliana* stably expressing GFP-ABD2^7^. The transgenic tobacco BY-2 cell line was used to obtain the PPB images because of the high level of cell cycle synchrony that can be obtained using the DNA polymerization inhibitor aphidicolin, as previously reported^26^. Images of cortical microtubules at G1 phase and PPB at late G2 phase were obtained using 7-day-old cells (a stationary phase) and cell-cycle-synchronized cells 6­7 h after aphidicolin washout (a peak time for PPB)^47^, respectively. *A. thaliana* plants expressing GFP-ABD2 are commonly used to examine plant actin filaments^13^. The sterilized seeds were grown on solid half-strength Murashige and Skoog (MS) medium for 7 days in growth chambers set at 23.5 °C and a 16/8-h light/dark cycle using 85 µmol m^−2^ s^−1^ white lights, then the seedlings were immersed in half-strength MS liquid medium with DMSO or TIBA for 2 h. These plant materials were used to capture fluorescently-labeled cytoskeleton images on a CLSM equipped with a spinning-disk confocal scanner unit (CSU-X1, Yokogawa, Kanazawa, Japan) or a VAEM using a total internal reflection microscope (Olympus). YFP and GFP were excited using a 488-nm laser.

To capture actin filaments in *Aplysia* cells, the living growth cones of *Aplysia* bag cell neurons were observed with Pol-Scope^27,28^. Pol-Scope is a modified polarized light microscope designed to visualize birefringence retardation, and is useful for visualizing aligned actin filaments in unstained specimens^27^. We used the Pol-Scope time-lapse images that were used for the previous publication^27^.

To capture actin filaments in animal cells, the mouse/rat NG108‐15 cell line (in which cell differentiation is induced and actin filaments can be clearly visualized by TRITC-phalloidin staining) was used to capture the actin meshwork and bundles associated with filopodia in the same cells^48^. The STED images were obtained using a TCS SP8 STED 3X (Leica Microsystems, Wetzlar, Germany) with a 561-nm laser for excitation and a 660-nm laser with a donut beam for stimulated emission depletion^48^. The STED images were used for image processing and measurements without being deconvolved.

All microscopy and image acquisition settings were fixed so that the control and bundled cytoskeleton images could be compared.

### Image processing and measurements

All image processing was performed using ImageJ software^49^. The synthetic images simulating cytoskeleton bundling were made with an ImageJ macro (Supplemental Text 1). The macro parameters for this study were fixed as: Image Size = 100 × 100 pixels, Length of Filaments = 57 pixels, Bundle Coefficient = 20, Background Intensity = 0. Gaussian filtering and Gaussian noise addition were performed using ImageJ functions ‘Gaussian blur’ and ‘Add Specified Noise’, respectively. The microscope images were skeletonized before measurements of the intensity statistics were made using the ImageJ plug-in LpxLineExtract, as described previously^12^. CV and skewness were defined as

1 $$ CV = \frac{\sigma }{{\overline{i}}}, $$

2 $$Skewness = \frac{1}{N}\mathop \sum \limits_{i = 1}^{N} \left( {\frac{{i_{n} - \overline{i}}}{\sigma }} \right)^{3} ,$$

3 $$ \sigma = \frac{1}{N}\mathop \sum \limits_{i = 1}^{N} \left( {i_{n} - \overline{i}} \right)^{2} , $$

where *N*, $i_{n}$, and $\overline{i}$ are the cytoskeleton pixel numbers, intensity of a cytoskeleton pixel, and the mean intensity of cytoskeleton pixels, respectively. These image-intensity statistics were measured using our ImageJ macro (Supplemental Text 2). Mann–Whitney U-tests and Tukey–Kramer tests were performed using R (ver. 3.6.1; https://www.r-project.org/).

## Supplementary information

Supplementary Figures.

Supplementary Text 1.

Supplementary Text 2.
