# Supplementary material for: Coefficient of variation as an image-intensity metric for cytoskeleton bundling
Source: Sci Rep. 2020 Dec 21;10:22187. doi: 10.1038/s41598-020-79136-x (PMC7752905; doi:10.1038/s41598-020-79136-x)
Supplement: Supplementary file 1 — Supplementary Figures. [file 41598_2020_79136_MOESM1_ESM.pdf]

Supplementary Information

Coefficient of variation as an image-intensity metric for cytoskeleton bundling

Takumi Higaki[1*], Kae Akita[2], Kaoru Katoh[3]

[1]International Research Organization for Advanced Science and Technology, Kumamoto University, 2-39-1 Kurokami, Chuo-ku, Kumamoto, Japan

[2]Department of Chemical Biological Science, Faculty of Science, Japan Women's University, 2-8-1 Mejirodai, Bunkyo-ku, Tokyo, Japan

[3]Biomedical Research Institute, National Institute of Advanced Industrial Science and Technology (AIST), Tsukuba, Japan

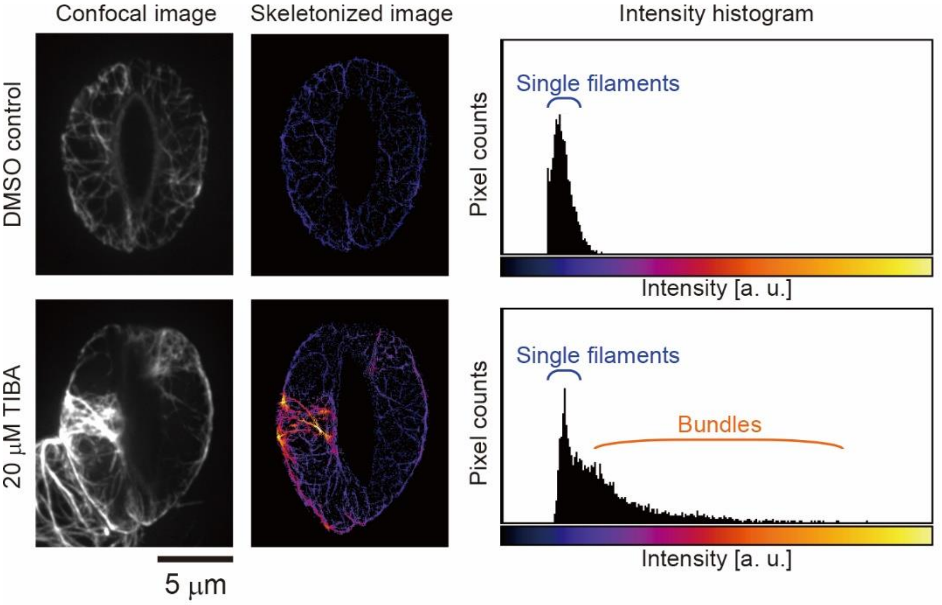

Supplemental Figure 1. Evaluation of cytoskeleton bundling using the skewness of the intensity distribution. Confocal images of GFP-labeled actin filaments in plant guard cells are shown (left). Bundling was induced by treatment with 2,3,5-triiodobenzoic acid (TIBA). Scale bar indicates 5 μm. The images were skeletonized to obtain the pixels representing the filaments, which are shown as the colored pixels (middle). The intensity histograms show the distribution of the pixels representing actin filaments (right). In the control (right top), most of the pixels represent low-intensity single filaments. When the filaments are bundled, pixels representing high-intensity bundles appear, in addition to those representing single filaments. The skewness of the intensity distribution increases when filaments are bundled.

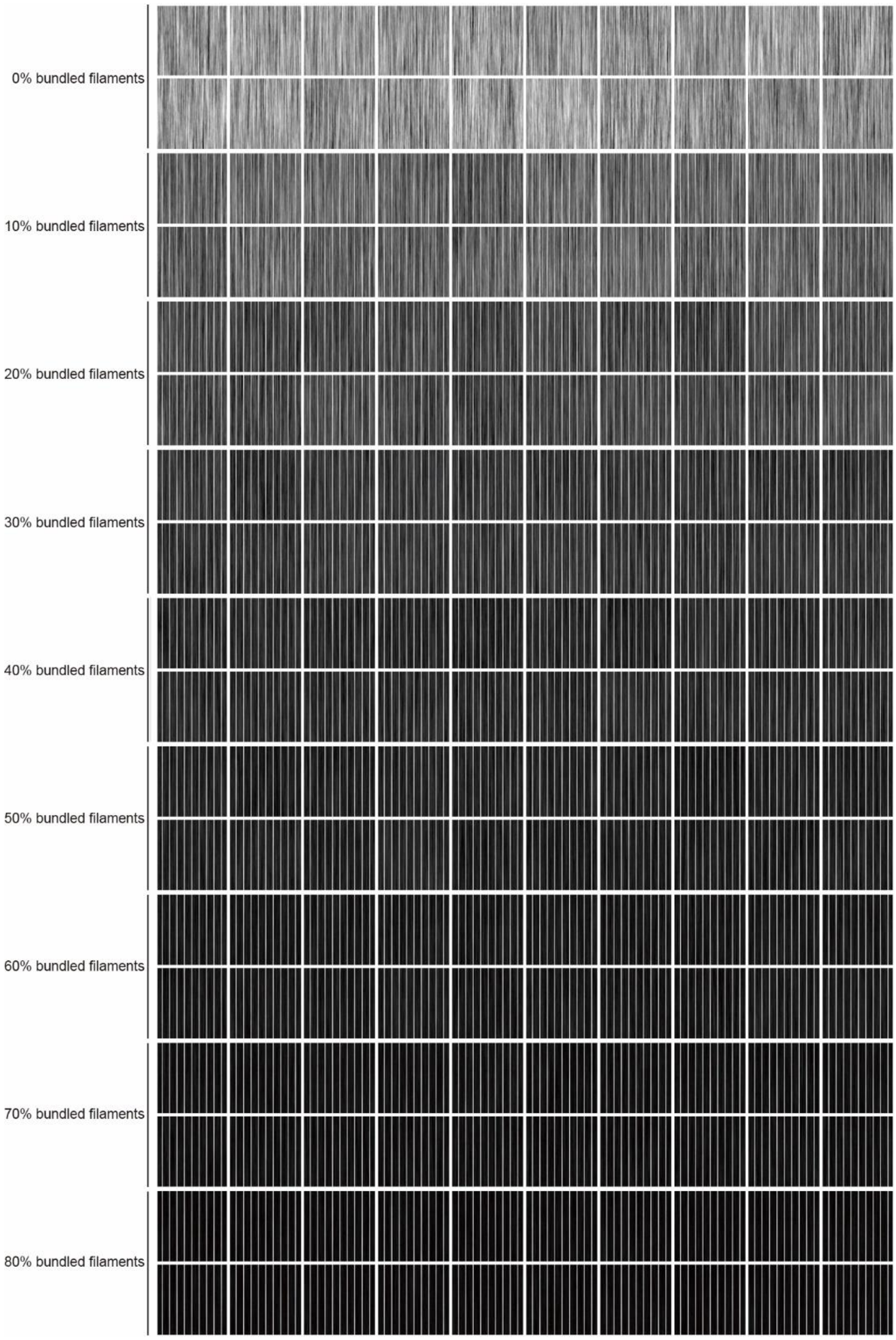

Supplemental Figure 2. Synthetic images of virtual cytoskeleton bundling. The images were created by adding filaments with constant intensity and length at random positions. To mimic bundles, the position of the filaments on the *X*-coordinate was restricted to 1/20 for different percentages of the

filaments (0%–80%). Images with a size of 100 × 100 pixels were used. N = 20.

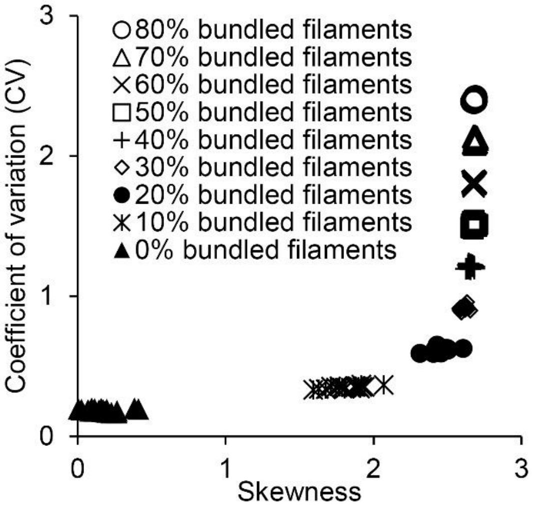

Supplemental Figure 3. A scatter plot of skewness and CV for the synthetic images of virtual cytoskeleton bundling. The image dataset shown in Supplemental Figure 2 was used. N = 20.

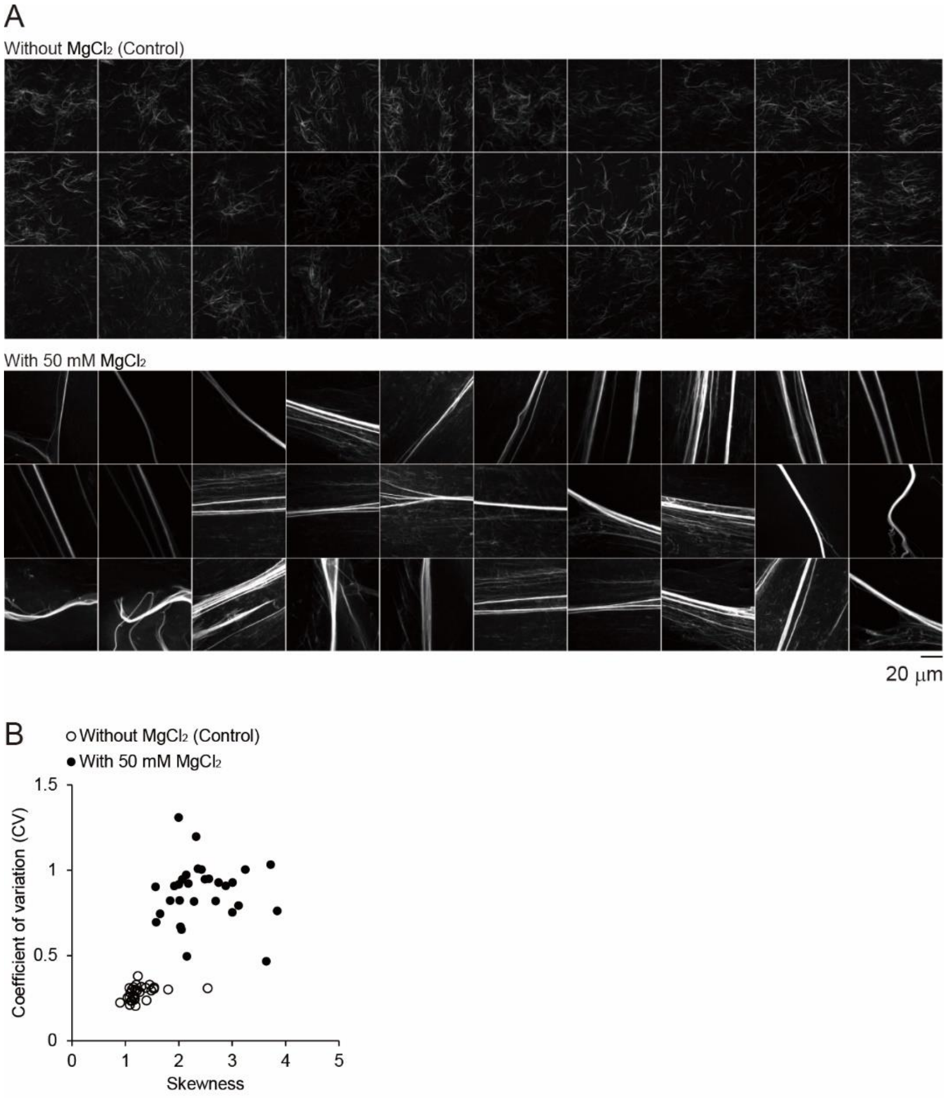

Supplemental Figure 4. Dataset of the CLSM images of *in vitro* actin filaments used in this study. (A) All CLSM images of the ATTO 390-labeled actin filament solution without (top) or with the addition of 50 mM $MgCl_2$ (bottom). Images with a size of 512 × 512 pixels were used. Scale bar indicates 20 μm. N = 30. (B) Scatter plot of skewness and CV.

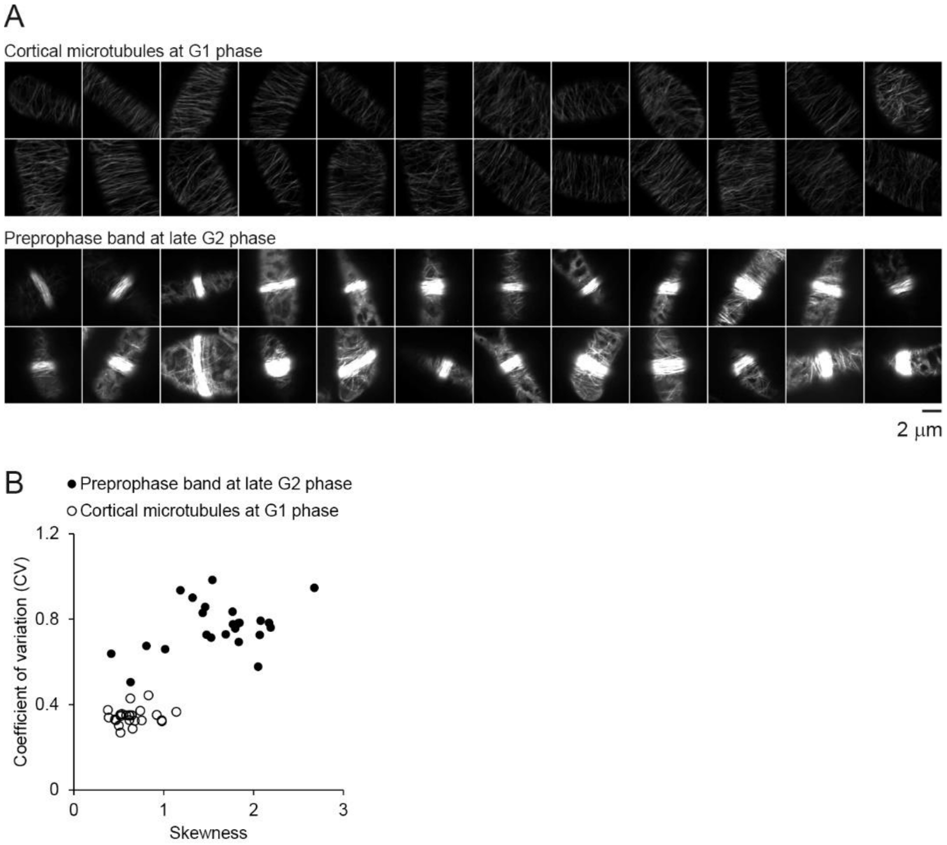

Supplemental Figure 5. Dataset of the CLSM images of tobacco BY-2 cells used in this study. (A) All CLSM images of the YFP-tubulin-labeled microtubules in tobacco BY-2 cells. Cortical microtubules at the G1 phase (top) and in preprophase bands at the late G2 phase (bottom) are shown. Images of size 400 × 400 pixels were used. Scale bar indicates 2 μm. N = 24. (B) Scatter plot of skewness and CV.

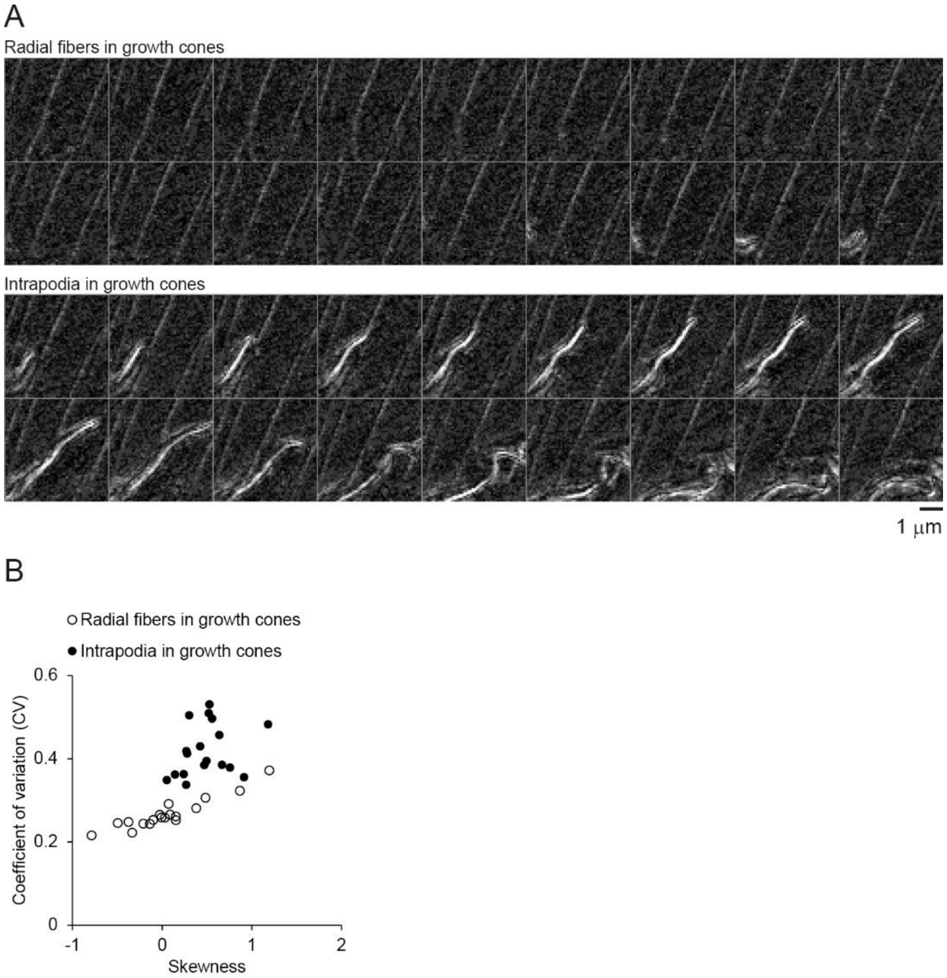

Supplemental Figure 6. Dataset of the Pol-Scope images of *Aplysia* bag cell neurons used in this study. (A) All Pol-Scope images of the radial actin fibers (top) and intrapodia composed of highly bundled actin filaments (bottom) in the growth cone are shown. Images with a size of 54 × 54 pixels were used. Scale bar indicates 1 μm. N = 18. (B) Scatter plot of skewness and CV.

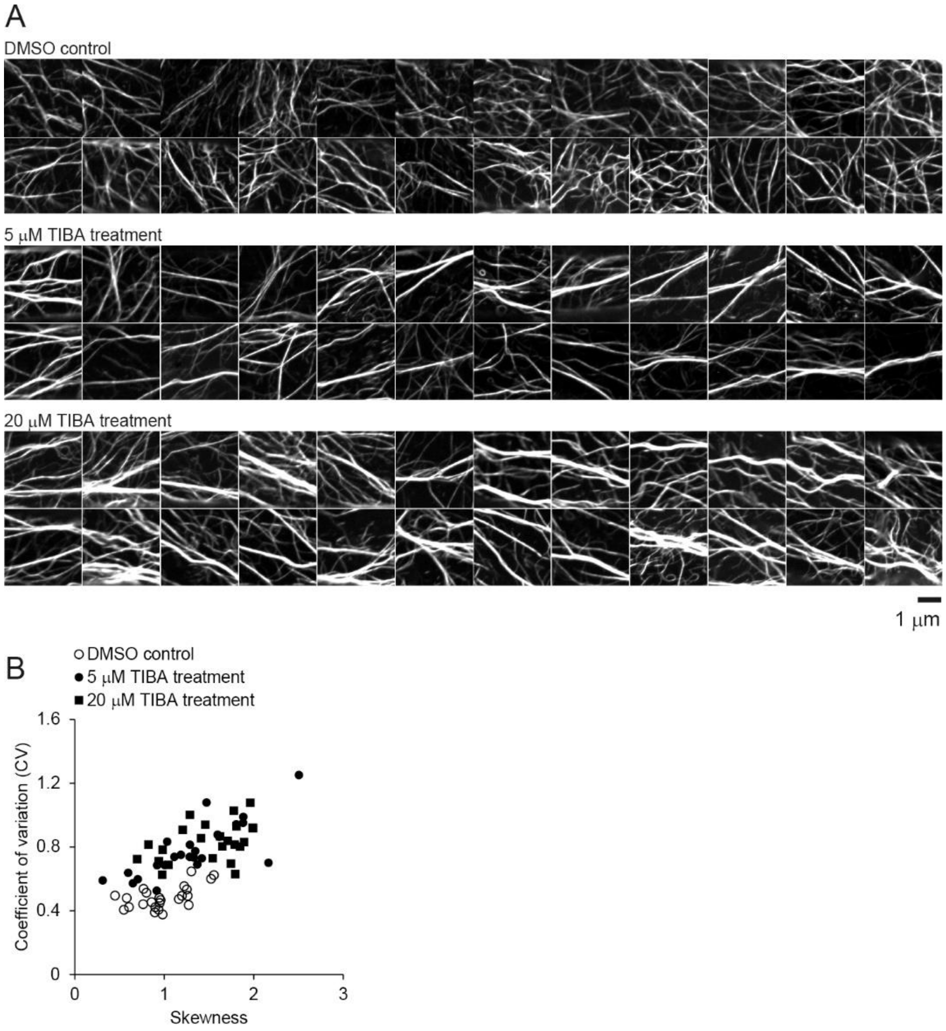

Supplemental Figure 7. Dataset of CLSM images of *A. thaliana* hypocotyl cells used in this study. (A) All CLSM images of the GFP-ABD2-labeled actin filaments in hypocotyl cells of *A. thaliana* plants. Actin filaments treated with DMSO (control; top), 5 μM (middle), and 20 μM TIBA, an actin filament bundling agent (bottom), are shown. Images with a size of 167 × 167 pixels were used. Scale bar indicates 1 μm. N = 24. (B) Scatter plot of skewness and CV.

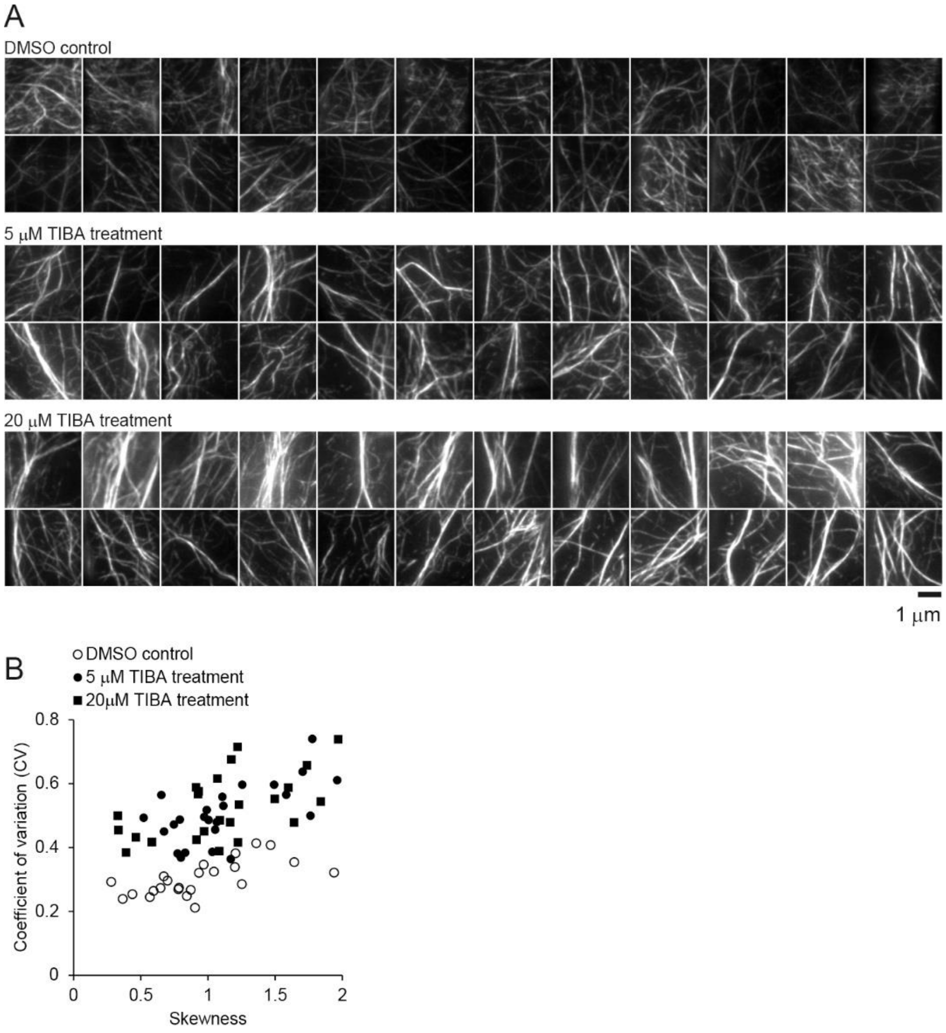

Supplemental Figure 8. Dataset of the VAEM images of *A. thaliana* hypocotyl cells used in this study. (A) All VAEM images of the GFP-ABD2-labeled actin filaments in hypocotyl cells of *A. thaliana* plants. The actin filaments treated with DMSO (control; top), 5 μM (middle), and 20 μM TIBA, an actin filament bundling agent (bottom) are shown. Images with a size of 100 × 100 pixels were used. Scale bar indicates 1 μm. N = 24. (B) Scatter plot of skewness and CV.

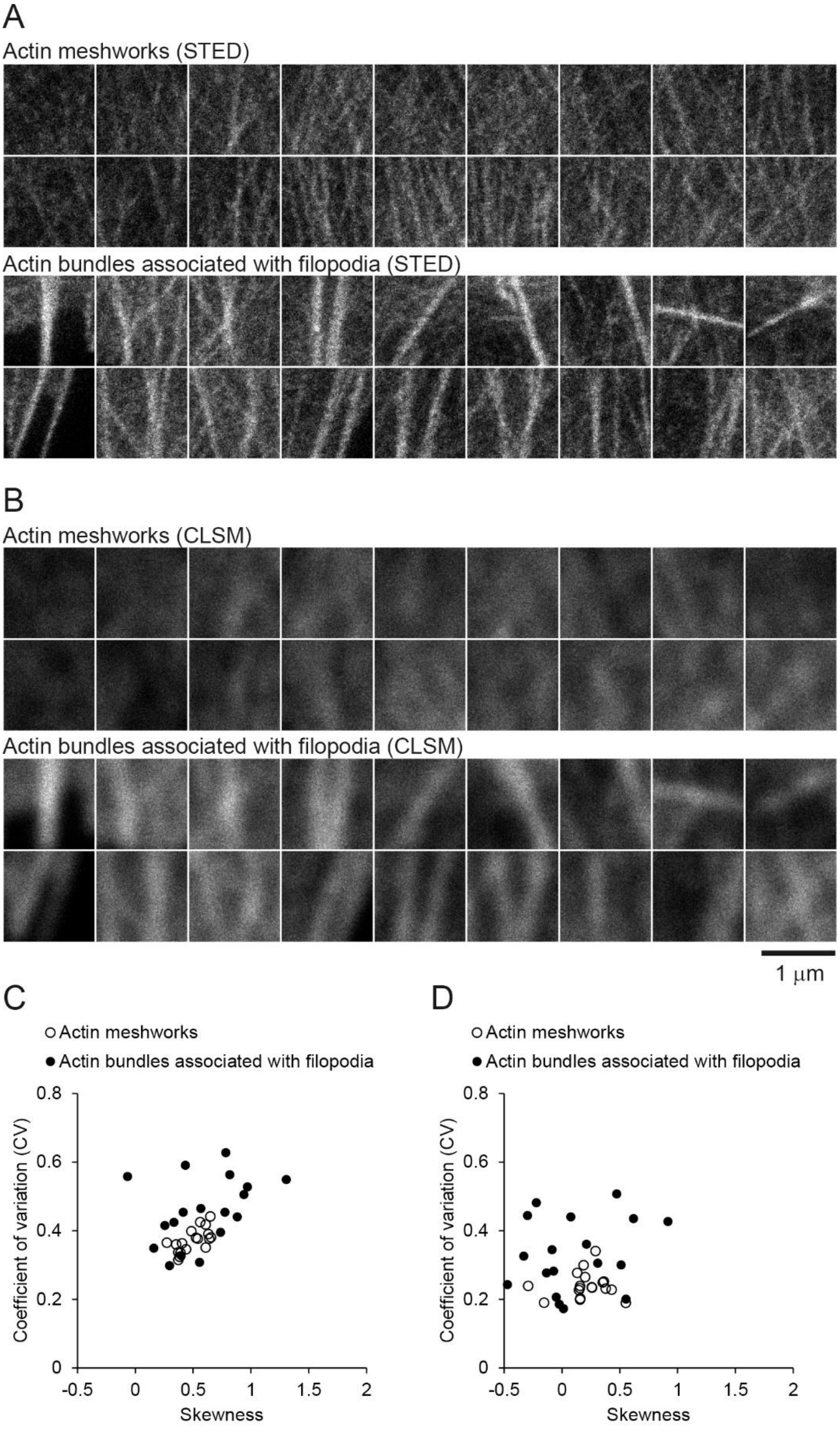

Supplemental Figure 9. Dataset of STED and CLSM images of the mouse/rat NG108-15 used in this

study. (A, B) All images of the TRITC-phalloidin-labeled actin filaments in NG108-15 cells. STED images of the actin meshwork (top) and actin filament bundles associated with filopodia (bottom) are shown (A). CLSM images of the same regions are also shown (B). Images with a size of 100 × 100 pixels were used. Scale bar indicates 1 μm. N = 18. (C, D) Scatter plot of skewness and CV for STED (C) and CLSM images (D).
